# Deep pathomics: A new image-based tool for predicting response to treatment in stage III non-small cell lung cancer

**DOI:** 10.1371/journal.pone.0294259

**Published:** 2023-11-28

**Authors:** Lorenzo Nibid, Carlo Greco, Ermanno Cordelli, Giovanna Sabarese, Michele Fiore, Charles Z. Liu, Edy Ippolito, Rosa Sicilia, Marianna Miele, Matteo Tortora, Chiara Taffon, Mehrdad Rakaee, Paolo Soda, Sara Ramella, Giuseppe Perrone

**Affiliations:** 1 Research Unit of Anatomical Pathology, Department of of Medicine and Surgery, Università Campus Bio-Medico di Roma, Rome, Italy; 2 Anatomical Pathology Operative Research Unit, Fondazione Policlinico Universitario Campus Bio-Medico, Rome, Italy; 3 Radiation Oncology, Department of Medicine and Surgery, Università Campus Bio-Medico di Roma, Rome, Italy; 4 Radiation Oncology, Fondazione Policlinico Universitario Campus Bio-Medico, Rome, Italy; 5 Unit of Computer Systems and Bioinformatics, Department of Engineering, Università Campus Bio-Medico di Roma, Rome, Italy; 6 Department of Clinical Pathology, University Hospital of North Norway, Tromsø, Norway; 7 Department of Medicine, Brigham and Women’s Hospital, Harvard Medical School, Boston, Massachusetts, United States of America; 8 Department of Radiation Sciences, Radiation Physics, Biomedical Engineering, Umeå University, Umeå, Sweden; Taoyuan General Hospital, TAIWAN

## Abstract

Despite the advantages offered by personalized treatments, there is presently no way to predict response to chemoradiotherapy in patients with non-small cell lung cancer (NSCLC). In this exploratory study, we investigated the application of deep learning techniques to histological tissue slides (deep pathomics), with the aim of predicting the response to therapy in stage III NSCLC. We evaluated 35 digitalized tissue slides (biopsies or surgical specimens) obtained from patients with stage IIIA or IIIB NSCLC. Patients were classified as responders (12/35, 34.7%) or non-responders (23/35, 65.7%) based on the target volume reduction shown on weekly CT scans performed during chemoradiation treatment. Digital tissue slides were tested by five pre-trained convolutional neural networks (CNNs)—AlexNet, VGG, MobileNet, GoogLeNet, and ResNet—using a leave-two patient-out cross validation approach, and we evaluated the networks’ performances. GoogLeNet was globally found to be the best CNN, correctly classifying 8/12 responders and 10/11 non-responders. Moreover, Deep-Pathomics was found to be highly specific (TNr: 90.1) and quite sensitive (TPr: 0.75). Our data showed that AI could surpass the capabilities of all presently available diagnostic systems, supplying additional information beyond that currently obtainable in clinical practice. The ability to predict a patient’s response to treatment could guide the development of new and more effective therapeutic AI-based approaches and could therefore be considered an effective and innovative step forward in personalised medicine.

## Introduction

Lung cancer is the most common cancer worldwide. Non-small-cell lung cancer (NSCLC) accounts for almost 85% of all lung cancers, and is often diagnosed in advanced stages (stage III or IV) [1]. Thanks to the advent of precision medicine, there are now many therapeutic strategies available for patients with advanced-stage NSCLC (i.e., radiotherapy, chemotherapy, molecular target therapy, and immunotherapy), and it is possible to administer personalized therapy to each patient based on clinical stage and molecular testing [2, 3]. However, the balance between these treatments’ side effects and effectiveness is not completely satisfying. Despite considerable progress over recent years in the treatment of locally advanced NSCLC with combinations of immunotherapy and chemoradiation, about 50% of patients will die within 4 years [4]. Thus, there remains a need for strategies to better select and treat patients affected by advanced-stage NSCLC.

Over the last decade, Artificial Intelligence (AI) has emerged as a crucial paradigm for improving personalized medicine due to several significant factors: the increasing accessibility of medical data, the development of more sophisticated algorithms, and the expansion of computational capacity. In the era of “omics sciences”, artificial intelligence (AI) techniques have been tested using large amounts of data, with the aim of improving personalized medicine [5–7]. Indeed, this paradigm has been applied in various oncological-related tasks to improve clinical patient outcomes, e.g., to optimize the radiation therapy fractionation plan using the Deep Reinforcement Learning paradigm [8], to detect the lung cancer tumor mass in CT scan modalities using a combination of superpixel and active contour algorithms [9], or to predict radiotherapy treatment outcomes for NSCLC patients using a multimodal late fusion approach combining different modalities [10]. The interested readers may refer to [11] for a review of the work on AI for precision oncology.

On this ground, current research is examining new emerging applications of AI for analyzing morphological features. The rise of digital pathology makes it possible to scan tissue slides, converting them into a digital format (whole-slide imaging; WSI) [12]. With the use of machine learning, several studies have conducted analyses with the goal of supporting histological diagnosis of cancer and metastasis (computer-aided diagnosis; CAD) [12–16]. To our knowledge, few studies have investigated applications of AI techniques to identify new quantitative digital pathology markers, to support personalized clinical decision-making in cases of NSCLC [17–19]. In [17] the authors propose an end-to-end segmentation and feature extraction pipeline to identify and morphologically characterize both tumor nuclei and tumor cytoplasm within histopathology images. These extracted features feed classical machine learning methods to distinguish shorter-term from longer-term survivors. In [18] the authors aimed to predict disease recurrence in early-stage NSCLC from digitized histopathology slides, proposing a traditional supervised classification model involving the most predictive morphological features extracted manually. In [19] the authors developed a tumour region shape-based prognostic model to predict survival outcomes in lung adenocarcinoma patients. The model consists of a deep neural network to automatise tumour region recognition, followed by the extraction of shape and boundary features. The main drawback of these works lies in their reliance on a substantial pre-processing phase, as the features necessitate manual extraction rather than being extracted automatically.

In this regard, convolutional neural networks (CNNs) have been widely used for visual tasks, because they can successfully learn automatic features, thereby avoiding the use of handcrafted features [20–22]. Moreover, deep pathomics could be used as a predictive and prognostic tool based on histological digitalized images [23].

In our present study, we tested whether deep pathomics analysis could be used to predict target volume reduction during chemoradiation treatment of stage III NSCLC.

## Materials and methods

### Patients

We selected a cohort of 35 patients affected by stage IIIA/B NSCLC (according to the Union of International Cancer Control, TNM Classification of Malignant Tumours - 7th edition) who were consecutively diagnosed from November 2012 to July 2014. Tumor tissue slides were obtained at the time of diagnosis, before treatment—including 31 (88.6%) small biopsies, and 4 (11.4%) surgical specimens. All samples were collected at the Pathology Unit of Campus Bio-Medico University Hospital Foundation, linked to a patient ID and analyzed anonymously.

We recorded the patients’ age at diagnosis, sex, and disease stage (IIIA or IIIB). All patients underwent treatment with concurrent chemoradiation according to the current standard of care. During treatment all patients underwent weekly chest CT simulations without contrast medium to assess tumor shrinkage and acute toxicity. These CT scans were evaluated by two radiation oncologists, who independently assessed target volume reduction, considering both primary site and lymph node metastasis. Therefore, each physician was able to judge whether reduction was 1) present and clinically significant (which could also have meant that the reduction did not occur over a predetermined percentage but the area where it occurred could reduce the dose to the lung parenchyma), 2) present and clinically non-significant, or 3) absent. Patients with present and clinically significant target volume reduction were defined as “responders”, while those in whom reduction was clinically non-significant or absent were classified as “non-responders”. In cases where the physicians agreed on the first category, contrast-enhanced CT was performed to better visualize node reduction, and a new target volume was delineated [24]. Using these definitions, 12 patients were classified as “responders” and 23 as “non-responders”.

The study protocol was approved by the Ethical Committee of Campus Bio-Medico University on October 30, 2012, and registered at ClinicalTrials.gov on July 12, 2018 with identifier NCT03583723 after an initial exploratory phase. A written informed consent was obtained in all patients.

### Data curation

The data curation phase orchestrates the organization of high-resolution Whole Slide Images (WSIs) derived from tissue biopsy sections, rendering them suitable for subsequent learning and classification phases through a meticulously constructed data pipeline.

**Fig 1:**
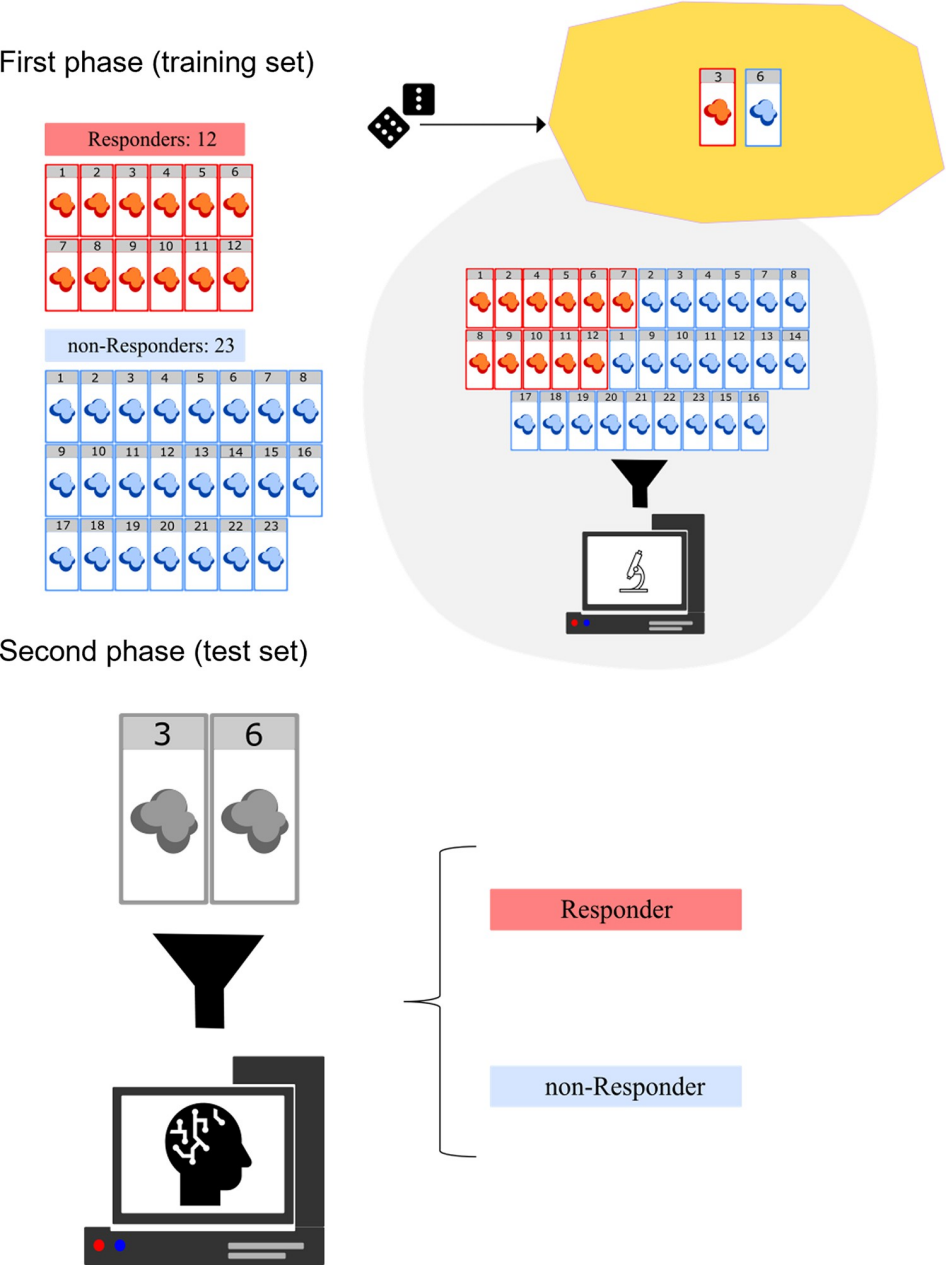
During the first phase, the data modeling randomly selected two tissue slides as the test set: One from the responder group (red tissue slides), and the other from the non-responder group (blue tissue slides). In the figure, slides 3 and 6 were selected as the test set from the responder and non-responder groups, respectively. The 33 remaining patients were used as the training set, to feed the machine learning system. All features from the 33 patients were extracted and analyzed to train the artificial intelligence. During the second phase, the convolutional neural networks (CNNs) ignored the belonging group of the two patients from the test set (gray tissue slides), and classified them as responders or non-responders based on their similarity to the training set tissue slides.

During the data collection process, the Pathomics dataset remains in a state of dynamic expansion with the addition of new samples. Each new sample traverses the data modeling component, wherein it is meticulously structured according to the patient ID. This arrangement ensures its accessibility for subsequent model learning and testing phases. Given the modest extent of the current dataset, and driven by the imperative to attain classifier generalization and unbiased outcomes, the data modeling component partitions the dataset through a patient-wise random sampling mechanism. Consequently, for each experimental iteration, the images are first grouped by patient, so that there is no possible overlap of the same patients between training and test sets, and then a leave-two patient-out cross validation was performed. Specifically, in each run, two patients (one per group) were assigned to the test set, and the other 33 patients to the training set. This stratagem guarantees that the crops of a particular patient are confined exclusively to either the training set or the test set which, in turn, helps to avoid model overfitting during the training phase (Fig 1).

### Digital image analysis

**Fig 2:**
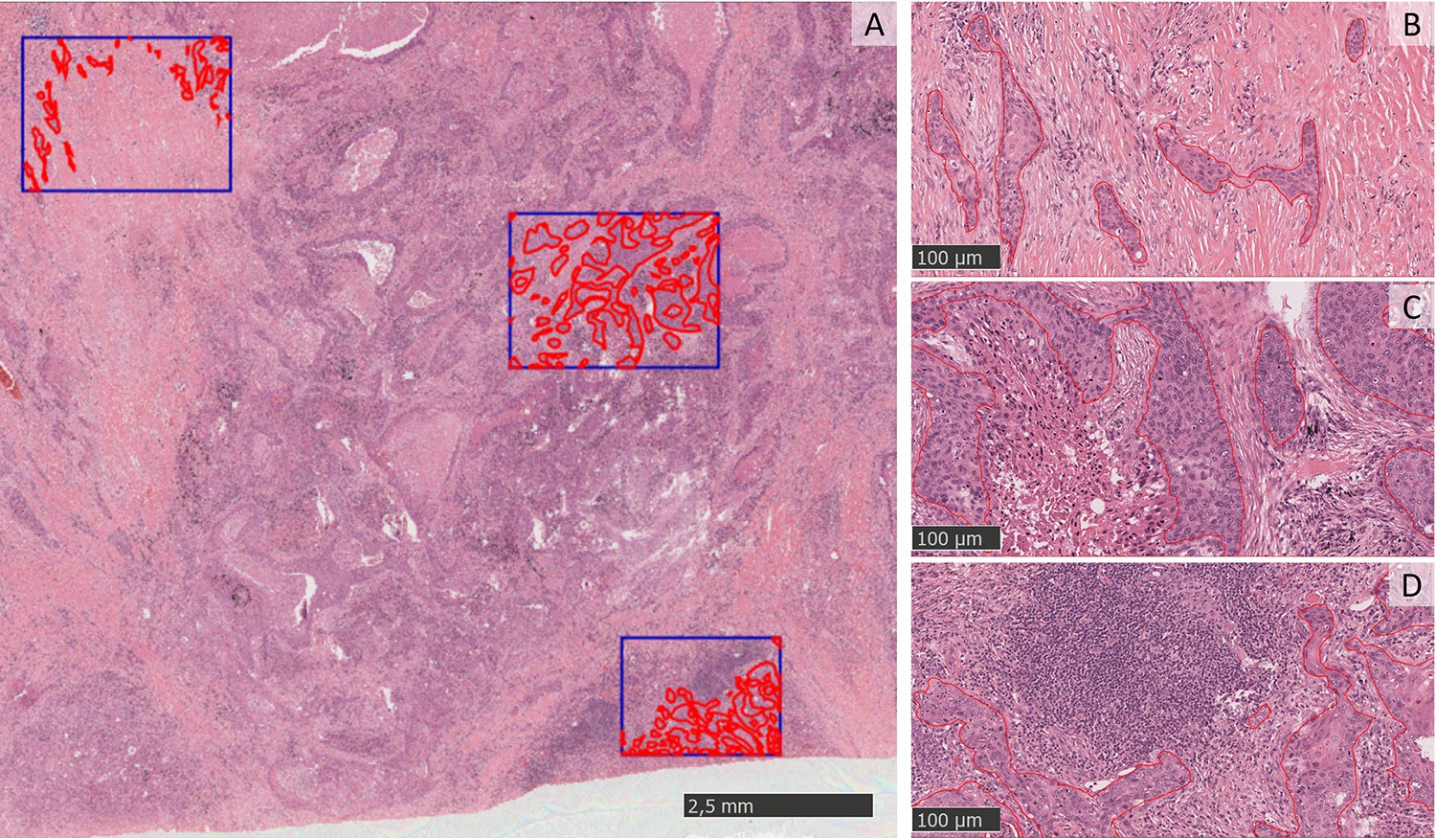
A) Tissue slide of a spindle cell carcinoma of the lung. Three regions of interest (ROIs) were selected (blue squares) based on tumor heterogeneity. B–D) Close-up views of tumor cells within desmoplastic fibrosis (B), areas of necrosis within tumor aggregates (C), and prominent inflammation surrounding tumor cells (D).

**Fig 3:**
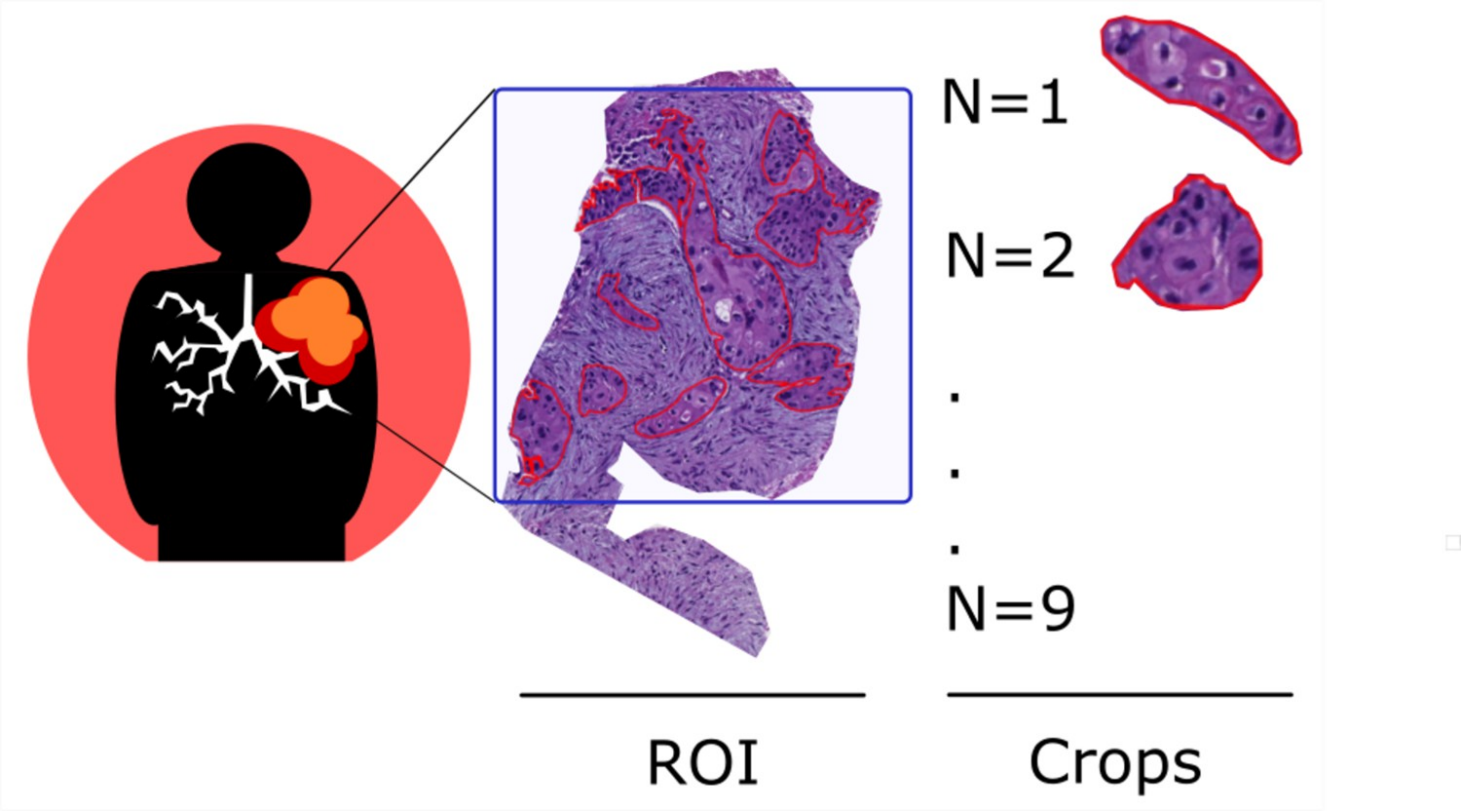
The sequence of crops and patches extracted from a single tumor. Each group of cancer cells is contoured, defining 9 crops (areas contoured in red) within a single region of interest (ROI; blue square).

Digital image analysis (DIA) of H&E-stained whole-slide images (WSIs) was performed from July 2019 to May 2020 using NanoZoomer 2.0 RT (Hamamatsu), at a magnification power of 20×, and a resolution of 0.5 μm per pixel. WSIs were loaded on QuPath software, and re-evaluated by an expert lung pathologist who selected a representative number of tumor areas, called regions of interest (ROIs). Tumor areas within the ROIs were manually segmented, which entailed contouring the tumor edge, while avoiding fibrosis, necrosis, and histological artifacts. The contoured tumor areas were defined as “crops”. The number of crops depended on the sample size and on the selected ROI. In detail, in surgical specimens, we determined the ROI by considering the tumor heterogeneity, including representative areas with necrosis, fibrosis or inflammation (Fig 2); differently, in case of very small tissue biopsies, the whole tumor was countered, and the number of crops depends exclusively on the sample size. A total of 1303 crops of different shapes and sizes (424 ± 698 × 393 ± 548) were manually segmented. All crops were reshaped to a size suitable as input for the convolutional neural networks (CNNs) used in this work, and are presented in Fig 3. In particular, all RGB images were resized to 224 × 224 × 3, such that the input layer of the pre-trained nets did not have to be changed.

### Deep learning with convolutional neural networks

In the present study, we incorporated five Convolutional Neural Networks (CNNs) that had been pre-trained on the ImageNet dataset, including AlexNet [25], VGG (Visual Geometry Group) [26], MobileNet [27], GoogLeNet [28], and ResNet (Residual Network) [29]. The rationale behind utilizing the ImageNet dataset for pre-training is two-fold. Firstly, it enables us to gauge the transferability of knowledge acquired from disparate domains to the field of Pathomics. Secondly, this dataset furnishes an ample repository of intricate image attributes pertaining to diverse entities and subjects, thereby substantiating our conviction in the potential of these pre-trained networks to effectively extrapolate their feature extraction proficiencies to the novel task within the domain of Pathomics.

#### AlexNet

Originating from the foundational principles espoused by LeNet [30, 31], AlexNet encapsulates the rudimentary tenets of CNN architecture and extends them to a profoundly intricate and expansive network model. Moreover, it is accredited with pioneering the integration of techniques such as Rectified Linear Units (ReLU), Dropout, and Local Response Normalization within the context of CNNs [25]. The AlexNet architecture constitutes a seminal advancement in the realm of convolutional neural networks (CNNs), particularly tailored for image classification endeavors.

**Fig 4:**
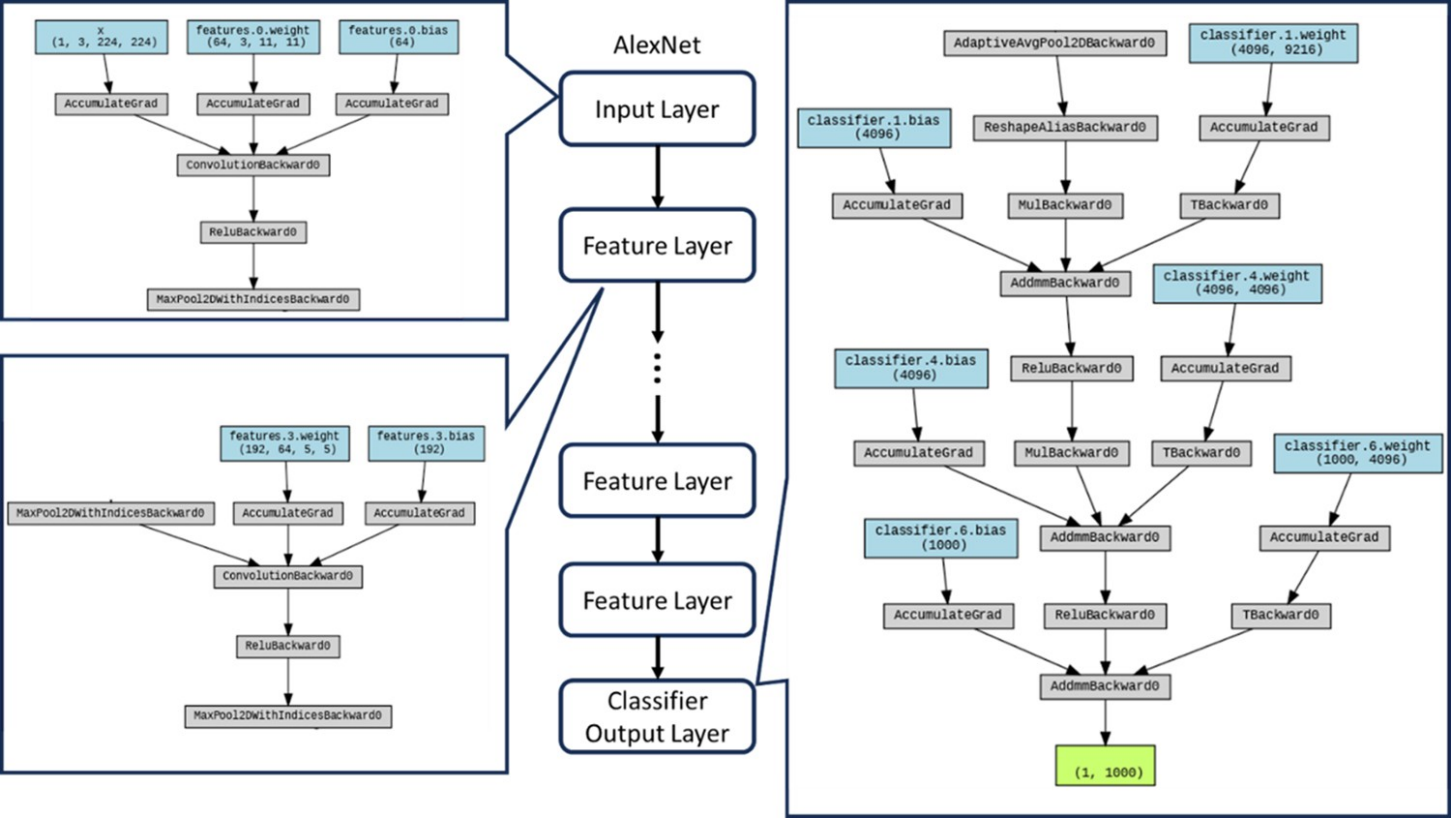
AlexNet structure with key workflow.

As shown in Fig 4, this architectural configuration encompasses several pivotal elements intrinsic to its design, contributing to its efficacy in addressing complex visual recognition tasks including **Input Layer**: The initiation of the AlexNet architecture involves the reception of input images, presented in the format of 224x224x3 pixels, signifying the dimensions of width, height, and the red-green-blue (RGB) color channels; **Convolutional Layers (Conv)**: A foundational stratum of the architecture is formed by an assemblage of convolutional layers, which serve as adept feature extractors from the input image domain; **ReLU Activation**: Adhering to each convolutional layer, the rectified linear unit (ReLU) activation function is judiciously applied. This non-linear activation fosters the network’s proficiency in discerning intricate patterns within the visual data; **Max Pooling Layers (Pool)**: Consequent to selecting convolutional layers, the integration of max pooling layers transpires. Their primary role encompasses a twofold purpose: ameliorating computational resource utilization and inducing a degree of translational invariance; **Local Response Normalization (LRN)**: A noteworthy inclusion within the AlexNet architecture is the employment of local response normalization (LRN). This facet of the architecture imparts a normative structure to neuron responses within localized regions, bolstering the network’s discriminative capacity toward specific salient features; **Fully Connected Layers (FC)**: The architecture prominently features a series of fully connected layers, adept at orchestrating the manipulation of abstracted high-level features furnished by preceding layers. This orchestration culminates in the generation of class predictions integral to image classification; **Softmax Layer**: The penultimate stage of the fully connected layers entails the implementation of the softmax activation function. Its application culminates in the derivation of probabilistic estimates across different classes based on the learned features; **Output Layer**: The final stratum of the architecture culminates in the provision of comprehensive classification results. These outcomes ascertain the assigned class identity for the input image under scrutiny.

Eminently noteworthy is the innovative utilization of techniques such as ReLU activation, dropout regularization, and data augmentation within the architecture, underpinning its seminal role in catapulting deep learning methodologies to the forefront of image classification paradigms. The cascading impact of AlexNet reverberates through the strides achieved in the expansive domain of visual recognition, validating the potential of CNNs to unravel intricate patterns latent within complex visual data.

#### VGG

Building upon the framework of AlexNet, VGG introduces refinements by augmenting the nonlinear separability through the amplification of network depth, simultaneously accompanied by a reduction in kernel dimensions aimed at bolstering computational efficiency [26].

**Fig 5:**
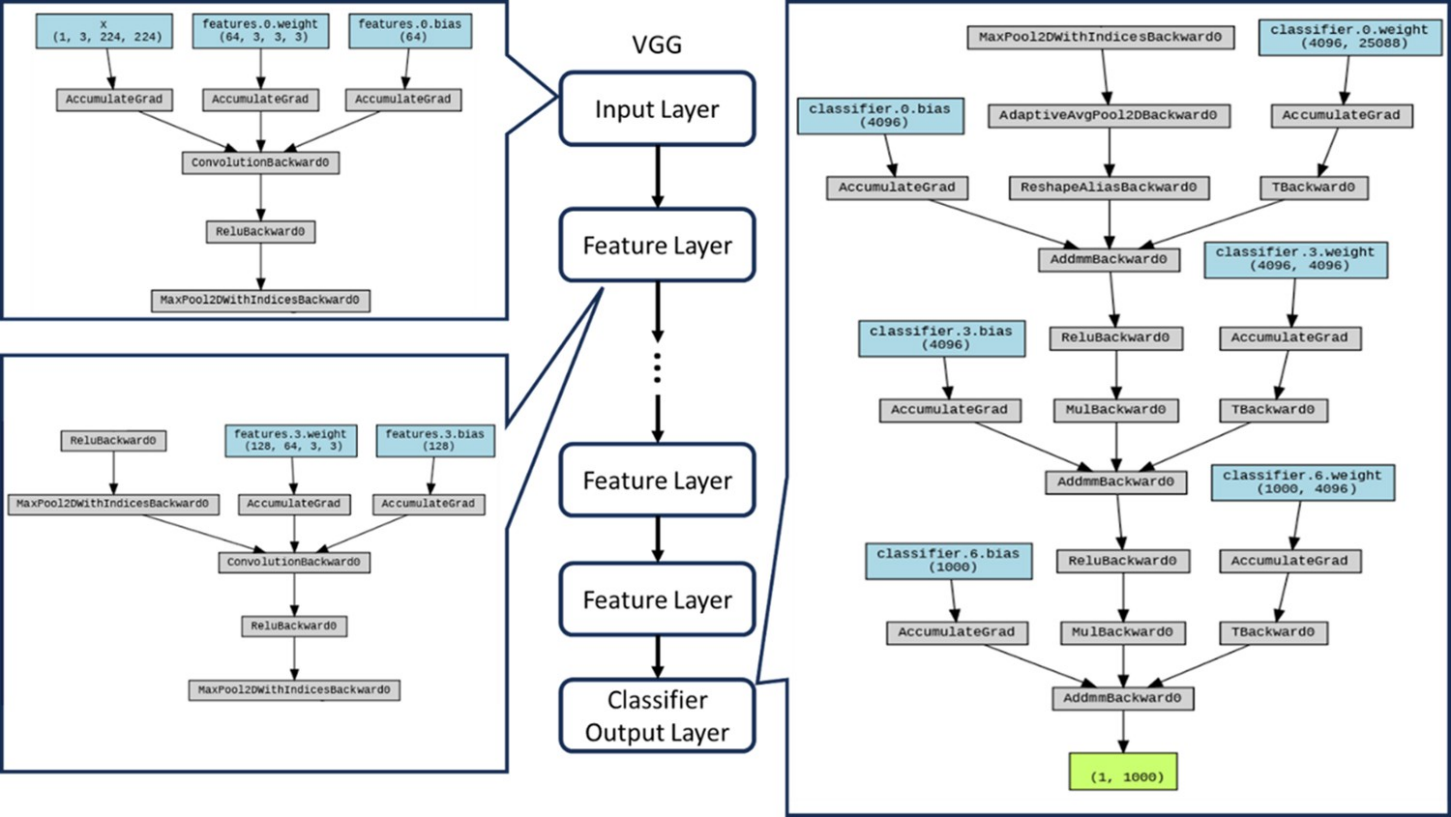
VGG structure with key workflow.

The VGG (Visual Geometry Group) architecture occupies a pivotal position in the trajectory of convolutional neural network (CNN) evolution, particularly tailored to address the intricate demands of image recognition tasks. As shown in Fig 5, central to its design are several discernible structural attributes, which collectively contribute to its efficacy and significance within the realm of deep learning, including **Homogeneity and Elegance**: The VGG architecture distinguishes itself through its hallmark attributes of structural uniformity and conceptual elegance. Characterized by a cohesive framework, it embodies a sequential progression of convolutional layers interspersed with max-pooling layers, collectively organized into distinct building blocks that recur throughout the network; **Convolutional Layers (Conv)**; **Rectified Linear Unit Activation (ReLU); Max Pooling Layers (Pool)**; **Fully Connected Layers (FC)**; **Softmax Activation; Output Layer; Terminal Output Stratum:** The final stratum within the architecture precipitates the ultimate outcome–a conclusive classification output. This output pertinently identifies and assigns the appropriate class label to the subject input image.

Noteworthy for its profound depth and standardized construct, VGG architecture is instrumental in delineating the implications of network depth in discerning intricate image features. Its structural coherence and interpretability amplify its role in contributing to the foundational tenets of CNN design and comprehension.

#### MobileNet

In a distinct vein, MobileNet embarks on a streamlined architectural trajectory, wherein the utility of depth-wise separable convolutions is harnessed to actualize lightweight deep neural networks [27]. While architectures like AlexNet and VGG achieve heightened training efficacy through depth augmentation, it is imperative to acknowledge the concomitant proliferation of adversarial phenomena encompassing overfitting, vanishing gradients, and exploding gradients.

The MobileNet architecture represents a pivotal advancement within the realm of convolutional neural networks (CNNs), distinctly tailored to address the exigencies of computational efficiency while retaining competitive performance in diverse computer vision tasks. The architecture’s distinctive structural attributes are underpinned by a strategic integration of innovative design principles, collectively contributing to its remarkable efficiency and effectiveness.

**Fig 6:**
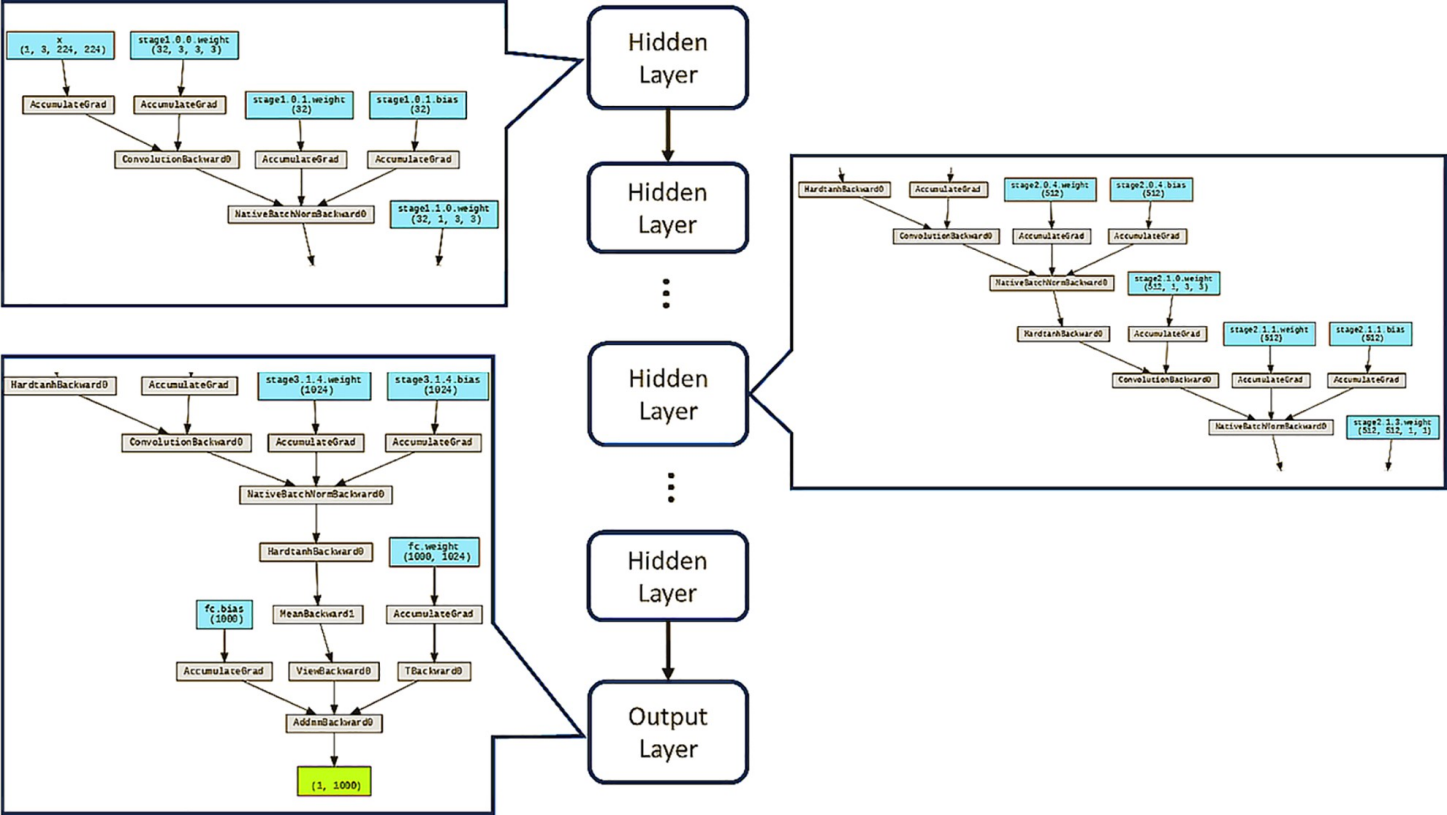
MobileNet structure with key workflow.

The main structure of MobileNet is shown in Fig 6, and its noteworthy features include **Depthwise Separable Convolutions**: A hallmark characteristic of MobileNet resides in the strategic implementation of depthwise separable convolutions. This paradigmatic approach partitions conventional convolutions into two distinct stages: depthwise convolutions, which act upon individual input channels, succeeded by pointwise convolutions, orchestrating the synthesis of depthwise convolution outputs through 1x1 convolutions. This segregation profoundly mitigates computational complexity, inducing a notable reduction in parameters and computational workload relative to conventional convolutional layers; **Parameter Efficiency through Pointwise Convolutions**: A cardinal tenet of MobileNet’s architecture revolves around parameter efficiency, prominently facilitated by the strategic employment of 1x1 pointwise convolutions. These convolutions harmonize information across channels while concomitantly diminishing dimensionality. This judicious information fusion precipitates the network’s ability to extract salient features using a parsimonious parameter count, rendering it particularly amenable to scenarios characterized by constrained computational resources; **Width Multiplier and Resolution Reduction**: A parameter termed the "width multiplier" emerges as a key facet of MobileNet’s architecture. By modulating the number of channels across layers, this multiplier offers a versatile knob for achieving a trade-off between model size and performance. Simultaneously, MobileNet introduces a "resolution reducer" facet, thereby further augmenting resource management through the manipulation of input resolution; **Bottleneck Architectures**: MobileNet innovatively incorporates bottleneck architectures, adeptly fusing 1x1 pointwise convolutions with 3x3 depthwise convolutions. This amalgamation engenders a transition from higher-dimensional spaces to lower-dimensional domains, culminating in a judicious balance between feature richness and computational load; **Global Average Pooling**: Departing from the convention of fully connected layers, MobileNet espouses the utility of global average pooling as an instrument of dimensionality reduction. By computing the average value for each channel across spatial dimensions, this operation begets a succinct yet representative feature representation; **Final Classification Layer**: The culminating facet of MobileNet’s architectural composition manifests in a softmax classification layer. This stratum bequeaths class probabilities contingent on the features distilled through the course of the network’s operation.

The structural innovations inherent within MobileNet collectively signify its paramountcy in effecting a paradigm shift within CNN architecture. By ushering in a new era of computational efficiency through innovative convolutional operations and parameter-efficient strategies, MobileNet stands as a harbinger of transformation in the field, adeptly addressing the computational constraints imposed by contemporary deep learning applications.

#### GoogLeNet

In response to these quandaries, GoogLeNet [28] introduces a novel structural paradigm characterized by the preservation of network sparsity. This approach harnesses the computational prowess of densely populated matrices to extract nuanced features while upholding computational resource efficiency.

**Fig 7:**
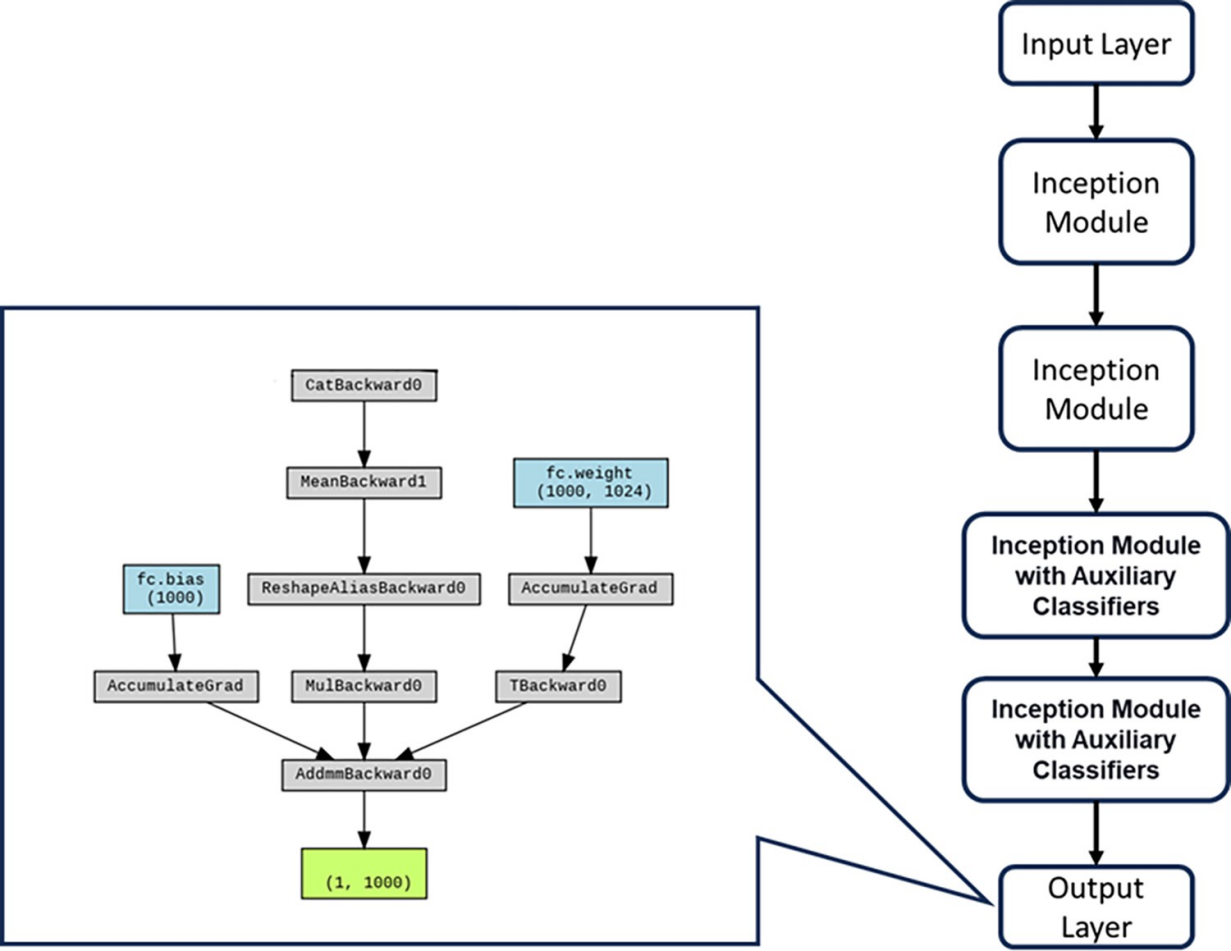
GoogLeNet structure with key workflow.

**Fig 8:**
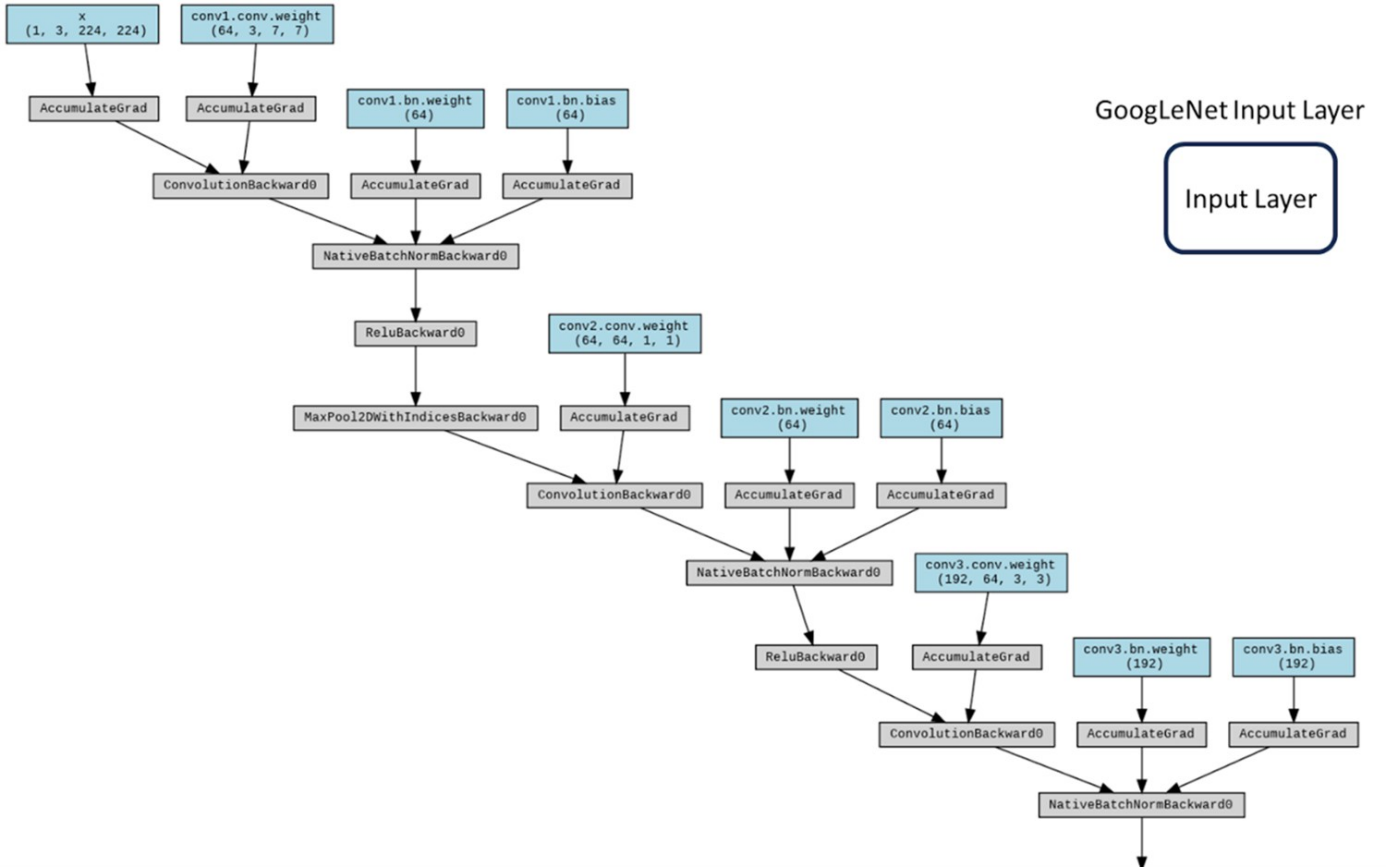
GoogLeNet input layer.

As shown in Fig 7, the GoogLeNet architecture, also known as Inception, represents a seminal breakthrough in convolutional neural network (CNN) design, prominently acclaimed for its innovative structure that prioritizes both depth and computational efficiency. The architecture’s hallmark feature is its inception modules, which foster the simultaneous extraction of features at varying scales, thus augmenting the network’s capacity for intricate pattern recognition. Structurally, GoogLeNet embodies a profound departure from traditional linear architectures with its featuring elements including **Inception Modules**: At the crux of GoogLeNet’s design lies the inception module, an ingenious assemblage of convolutional layers of distinct filter sizes and max-pooling operations. This amalgamation enables the extraction of features at different spatial hierarchies, effectively capturing fine-grained details alongside broader patterns. **Convolution for Dimensionality Reduction**: A pivotal insight within the inception module is the strategic employment of convolutions, designed not for spatial transformation but for dimensionality reduction. By reducing the number of input channels before applying more computationally intensive convolutions, GoogLeNet mitigates the computational burden, facilitating an efficient utilization of resources. **Parallel Convolutions**: GoogLeNet uniquely employs parallel convolutions within each inception module, harnessing the synergy of diverse filters to extract features at multiple scales and complexities. The concatenated outputs of these parallel paths encompass a comprehensive feature representation that contributes to improved discrimination and classification. **Inception with Auxiliary Classifiers**: GoogLeNet introduces auxiliary classifiers within intermediate layers of the network. These auxiliary classifiers contribute to the alleviation of the vanishing gradient problem, fostering more efficient and stable training. Furthermore, they serve as regularizers, promoting the acquisition of richer feature representations. GoogLeNet Input Layer is shown in Fig 8.

The distinctiveness of GoogLeNet lies in its ability to balance depth and computational efficiency while accommodating the increasing complexities of modern datasets. By pioneering the inception module concept and harnessing the potency of parallel operations, GoogLeNet significantly influenced subsequent CNN designs, making a resounding impact on the landscape of deep learning for image recognition tasks.

#### ResNet

ResNet [29] surmounts the quandary of network degradation through the incorporation of residual structures, ameliorating challenges such as gradient vanishing or explosion by means of normalized initialization and other methodological interventions. In conformity with our investigative design, the fundamental architectural blueprints of these networks were retained, albeit with the exception of the ultimate classification layer, which was tailored to accommodate a binary classification task.

**Fig 9:**
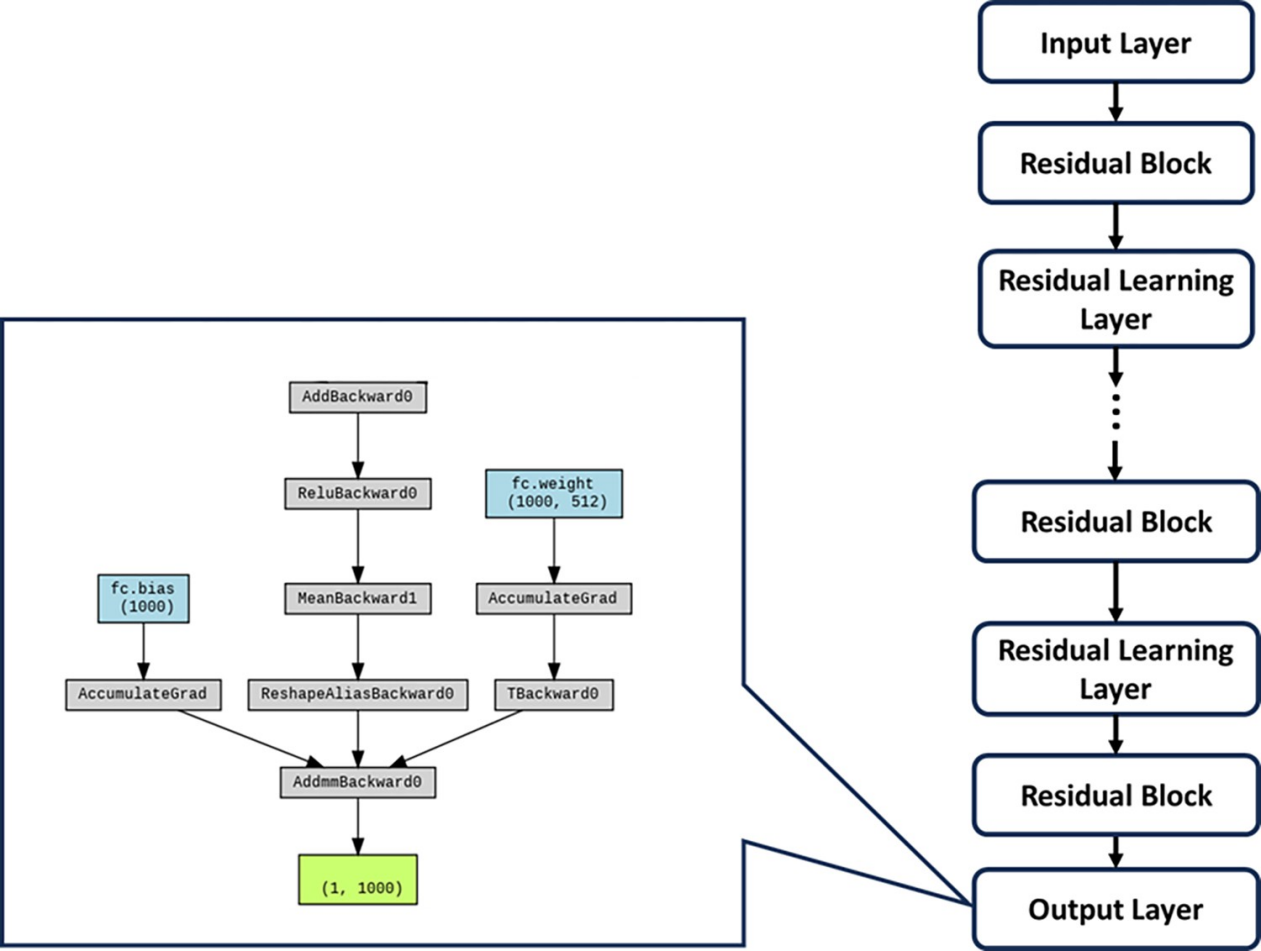
ResNet structure with workflow.

**Fig 10:**
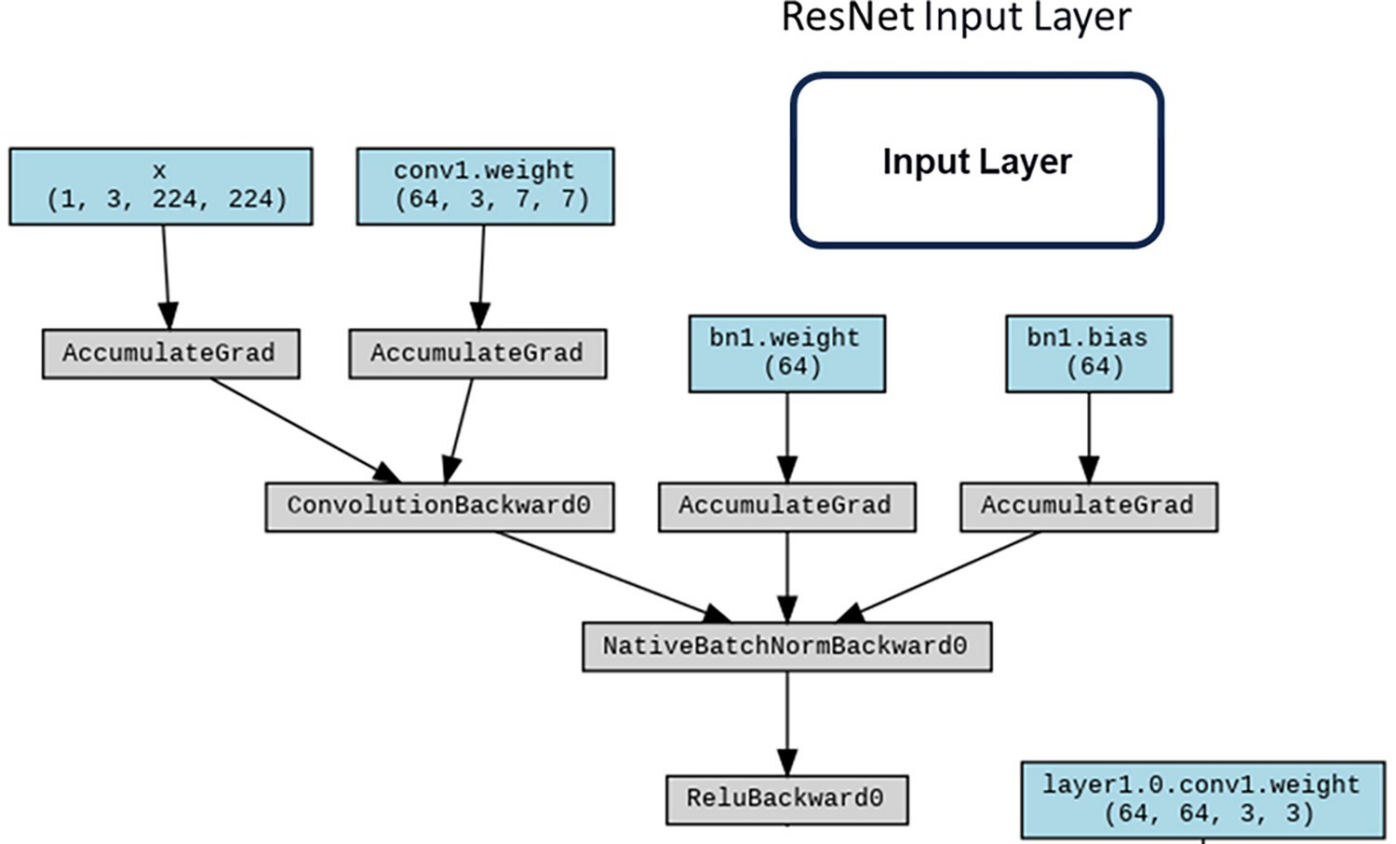
ResNet input layer.

**Fig 11:**
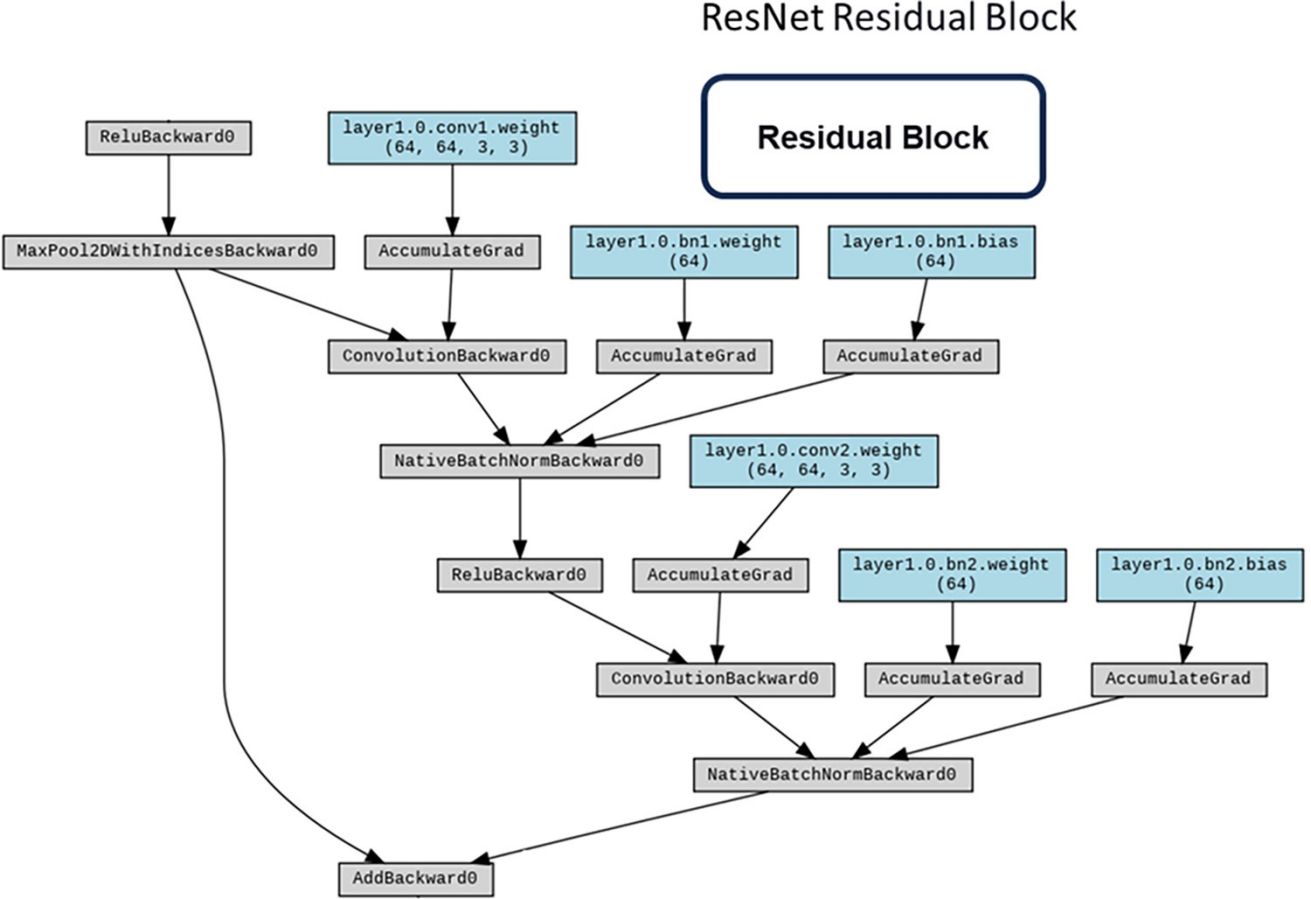
ResNet residual block.

**Fig 12:**
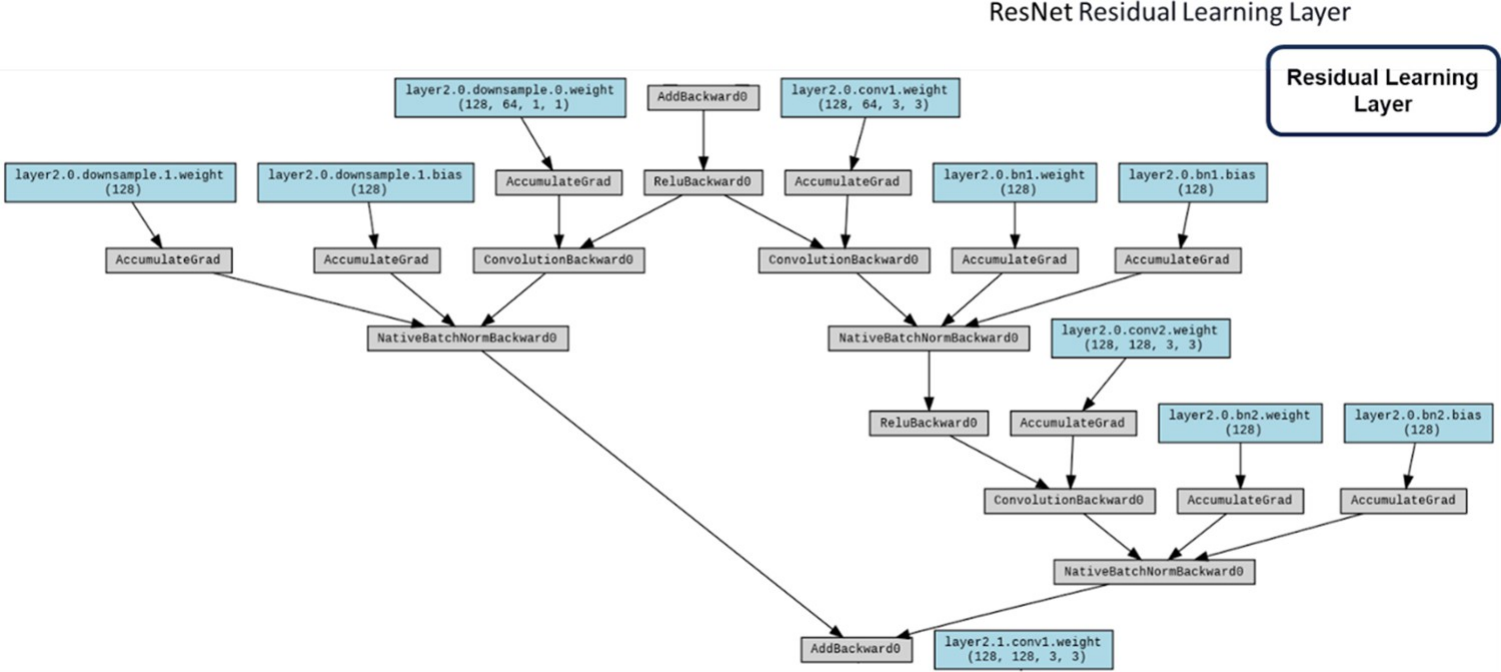
ResNet residual learning layer.

The ResNet architecture stands as a seminal advancement in the realm of convolutional neural networks (CNNs), specifically tailored to address the challenges posed by exceedingly deep networks. Figs 9 and 10 shows the main structure and the Input Layer of ResNet respectively. Rooted in the concept of residual learning, ResNet introduces structural modifications that facilitate the training of extremely deep networks while mitigating the vanishing gradient problem. The architecture’s core features are emblematic of its ability to accommodate unprecedented depth and foster improved feature learning including:

- **Residual Blocks**: A defining feature of ResNet architecture is the incorporation of residual blocks. These blocks consist of skip connections, or shortcuts, that enable the direct transfer of information from one layer to a subsequent layer, bypassing intermediate transformations. This residual mapping principle alleviates the vanishing gradient issue, enabling more effective training of deeper networks (Fig 11).
- **Identity and Projection Shortcuts**: Residual blocks come in two variants: identity shortcuts and projection shortcuts. Identity shortcuts maintain the input’s spatial and channel dimensions, while projection shortcuts introduce additional convolutional layers to adapt the dimensions, ensuring seamless merging of feature maps.
- **Bottleneck Architecture**: ResNet architecture also integrates a bottleneck architecture within residual blocks. This design comprises a sequence of 1x1, 3x3, and 1x1 convolutions, reducing computational load while maintaining feature richness.
- **Skip Connections for Deep Networks**: The core innovation of ResNet resides in its utilization of skip connections, enabling seamless propagation of gradients even through extremely deep networks. These connections facilitate the training of networks with hundreds of layers, which was previously infeasible due to the vanishing gradient problem.
- **Deep Residual Learning**: ResNet’s pivotal concept of deep residual learning posits that optimizing the residual mapping is easier than optimizing the original mapping. This insight serves as a foundational principle in architectural design and training methodology (Fig 12).

The potency of ResNet architecture lies in its adeptness at accommodating substantial network depth while ensuring effective training and feature learning. The innovative integration of skip connections and residual blocks signifies a paradigm shift in CNN design, permitting the construction of highly sophisticated models capable of handling intricate image recognition tasks. ResNet’s profound impact reverberates across the deep learning landscape, inspiring further developments in architectural sophistication and optimization techniques.

### Data analysis

CNNs were trained to obtain a prognostic network for patient classification using a transfer learning technique [32]. We used the five different CNNs described in the previous section, and all of them have been pre-trained on the ImageNet dataset: AlexNet, VGG, MobileNet, GoogLeNet, and ResNet. These CNNs were then trained at the crop level using the transfer learning technique [33], and we then used majority voting to assign the final prediction to a patient:

$$Op=\left\{ \begin{array}{l} \;Responder,\;if\;\frac{Na}{Nt}\geq 0.5\\ non\mathrm{Responder},\;otherwise \end{array}\right.$$

where *Na* is the total number of crops classified as responder, and *Nt* is the total number of tested crops per patient. This approach introduces a certain degree of redundancy with the aim of mitigating possible classification error at the crop level. The networks’ performances can be evaluated at both the patient and crop levels, offering insights into the system behavior at different granularity levels.

## Results

### Patients’ characteristics

The median patient age was 73 years (range, 52–87 years). Of the 35 patients, 21 (60%) received their diagnosis at stage IIIA, and 14 (40%) at stage IIIB. In terms of histological diagnosis, 23/35 (65.7%) samples were adenocarcinoma, while 12/35 (34.3%) were squamous cell carcinoma. During chemoradiation treatment, the mean target volume reduction observed was 39.8% (DS = 13.0%). Among the 35 patients, 12 (34.3%) were classified as responders, and 23/35 (65.7%) as non-responders. Target reduction was reached by 8/23 (34.8%) patients affected by adenocarcinoma, and by 4/12 (33.3%) patients affected by squamous cell carcinoma. Table 1 presents the patients’ characteristics.

**Table 1:** Patients’ characteristics.

Total = 35 (100%)	N (%)
**Responder**	
Yes	12 (34.3)
Not	23 (65.7)
**Age**	
Median, years	73
Range, years	52–87
**Histology**	
Adenocarcinoma	23 (65.7)
Squamous	12 (34.3)
**Stage**	
IIIA	21 (60.0)
IIIB	14 (40.0)
**Chemotherapy before chemoradiation**	
Yes	21 (60.0)
No	14 (40.0)
**Total radiotherapy dose**	
<60 Gy(200cGy/day)	12 (34.3)
≥60 Gy(200cGy/day)	23 (65.7)

### Pathomics analysis

**Fig 13:**
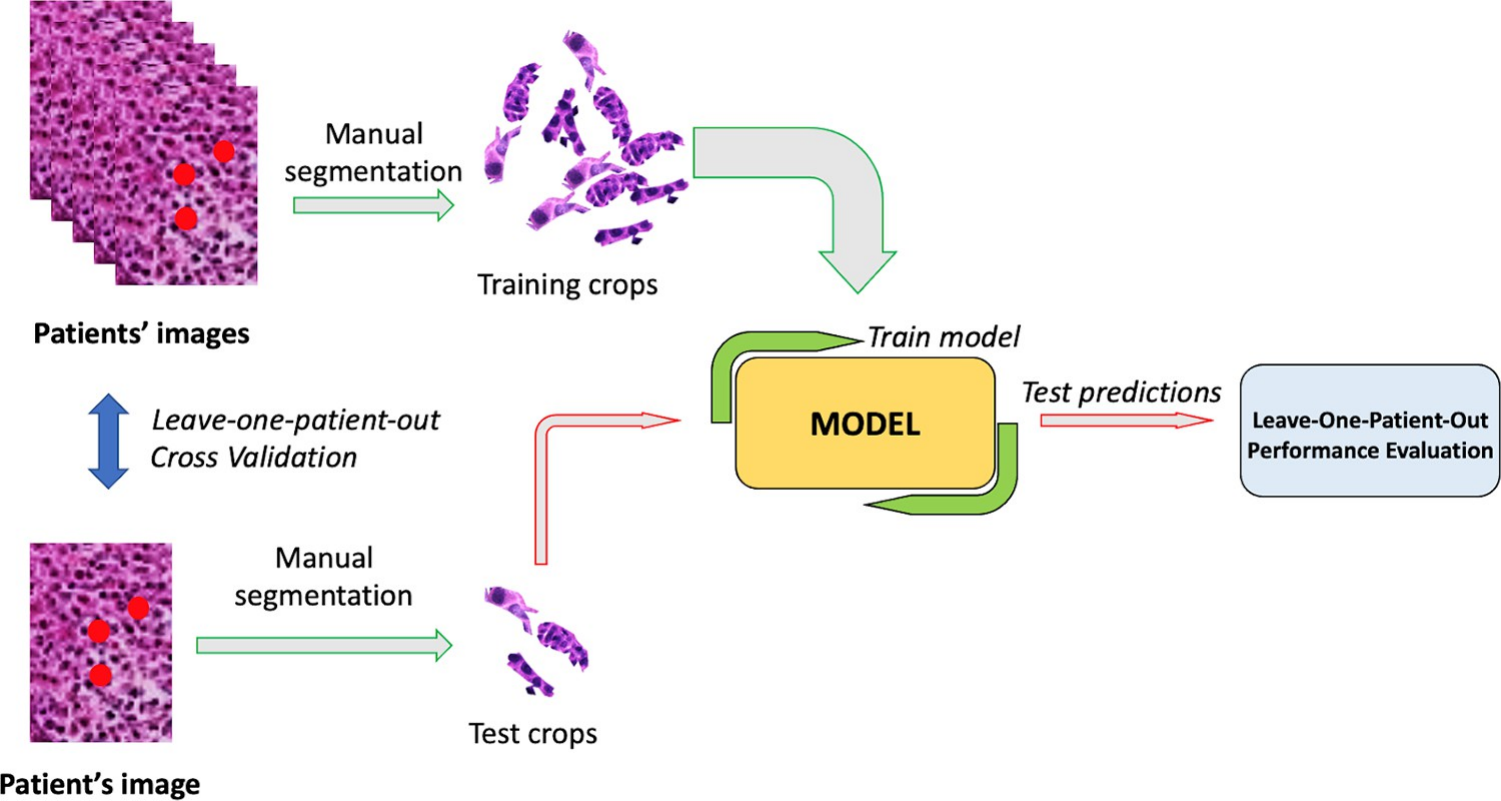
Depiction of the utilised pipeline. it consists of the following parts:
**Image acquisition:** several images are acquired for each patient;**Images segmentation:** each image undergoes a manual segmentation process by an experienced physician to extract only the pixels containing the cancer cells;**Model training:** the selected pre-trained model is fine-tuned on all the training crops belonging to the selected fold. The validation technique is the leave-one-patient-out and ensures that all the crops extracted from a patient can compose either the training or the test set, thus preventing the model from being biased;**Model test:** all the crops in the test set of the selected fold are analysed by the trained model, which returns a prediction. In order to maintain balance between classes each test set always consists of the crops from two patients coming with different labels;**Performance evaluation:** once the model has predicted the outcome of all crops from each test fold, a global performance evaluation step calculates the metrics needed to compare the models.

In this section we present the pipeline utilised to train and evaluate the performance of each model. The block scheme is depicted in Fig 13.

The described pipeline is then used on all the CNN models that let us obtain the results detailed in the next part.

Our analysis of CNNs showed that GoogLeNet globally achieved the best results, with accuracy of 0.78, F1 score of 0.76, and precision of 0.89. In detail, GoogLeNet correctly classified 8/12 responder patients, and 10/11 non-responder patients, with a true negative rate of 0.91 (specificity), and a true positive rate of 0.67 (sensitivity). Only 5/23 patients were misclassified by GoogLeNet—including 4 responder patients misclassified as non-responders, and 1 non-responder patient classified as a responder (Table 2).

**Table 2:** The performance of the convolutional neural network (CNN) architectures at the patient level, in terms of accuracy, F1 score, and precision.

	AlexNet	GoogLeNet	MobileNet	ResNet	VGG
**Accuracy**	0.74	0.78*	0.61^¶^	0.74	0.65
**F1 score**	0.73	0.76*	0.61	0.75	0.60^¶^
**Precision**	0.80	0.89*	0.64^¶^	0.75	0.75

* better results.

^¶^ worst results.

ResNet achieved 0.74 in accuracy, 0.75 in F1 score, and 0.75 in precision. Additionally, ResNet reached the best result in terms of sensitivity, correctly classifying 9/12 responder patients, yielding a true positive rate of 0.75. Moreover, ResNet detected 8/11 non-responder patients, reaching a true negative rate of 0.73. ResNet misclassified 6/23 patients—including 3 responder patients misclassified as non-responders, and 3 non-responder patients classified as responders.

AlexNet achieved 0.74 in accuracy, 0.73 in F1 score, 0.80 in precision, 0.67 in sensitivity, and 0.82 in specificity. In detail, AlexNet correctly classified 8/12 responders, and 9/11 non-responders. AlexNet misclassified 6/23 patients, including 4 responder patients misclassified as non-responders, and 2 non-responders misclassified as responders.

VGG achieved 0.78 in accuracy, and 0.79 in precision. Notably, VGG exhibited the worst F1 score (0.6) and sensitivity, correctly detecting only 6/12 responder patients, with a true positive rate of 0.5. On the other hand, VGG correctly classified 9/11 (81.8%) non-responder patients. VGG misclassified 8/35 patients, including 6 responder patients misclassified as non-responders, and 2 non-responder patients classified as responders.

MobileNet achieved 0.61 in F1 score, and obtained the worst results in terms of accuracy (0.6), precision (0.64), and specificity. MobileNet successfully classified 7/11 non-responder patients, yielding a true negative rate of 0.64, and correctly detected 7/12 responder patients, yielding a true positive rate of 0.58. MobileNet misclassified 9/35 patients, including 4 responder patients misclassified as non-responders, and 5 responders misclassified as non-responders.

Tables 3 and 4 summarizes the analysis of CNNs and it shows the results obtained by each model on the test set in terms of Confusion Matrix (CM), True Positive rate (TPr) and True Negative rate (TNr). S1 Table shows the classification category for each image by each network.

**Table 3:** The tables are the CMs obtained by each CNN in the test set.

**AlexNet**	*Predicted positive*	*Predicted negative*	**GoogLeNet**	*Predicted positive*	*Predicted negative*
*Real positive*	**8**	**4**	*Real positive*	**8**	**4**
*Real Negative*	**2**	**9**	*Real Negative*	**1**	**10**
**MobileNet**	*Predicted positive*	*Predicted negative*	**ResNet**	*Predicted positive*	*Predicted negative*
*Real positive*	**7**	**5**	*Real positive*	**9**	**3**
*Real Negative*	**4**	**7**	*Real Negative*	**3**	**8**
**VGG**	*Predicted positive*	*Predicted negative*			
*Real positive*	**6**	**6**			
*Real Negative*	**2**	**9**			

**Table 4:** The table reports the True positive rate (TPr) and the True negative rate (TNr).

Statistics	AlexNet	GoogLeNet	MobileNet	ResNet	VGG
**TPr**	0.67	0.67	0.58	0.75*	0.50^¶^
**TNr**	0.82	0.91*	0.64^¶^	0.73	0.82

* better results.

^¶^ worst results.

**Fig 14:**
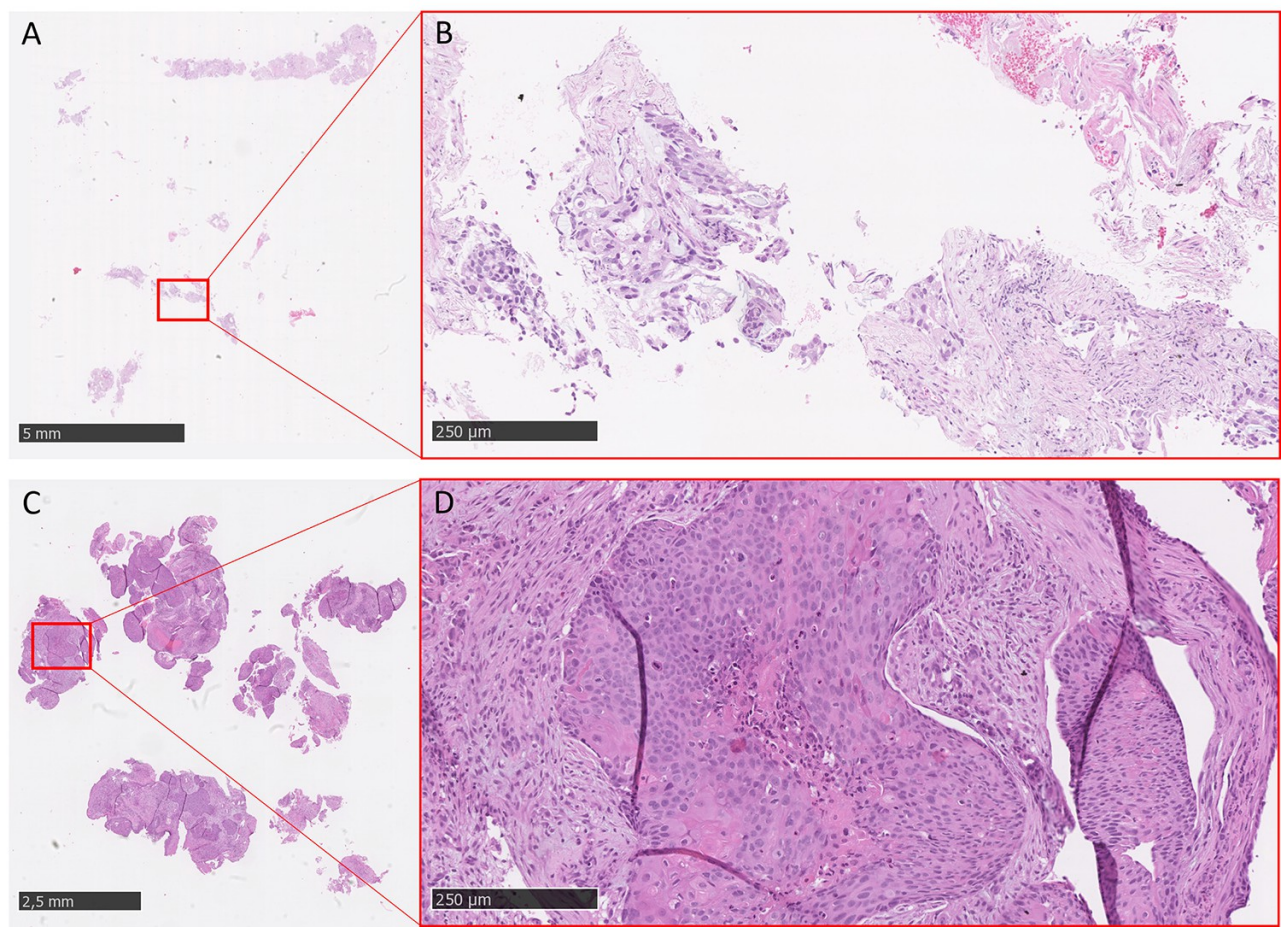
Two tissue slides that were failed by all the convolutional neural networks (CNNs). A) Whole-slide image (WSI) of a responder patient affected by adenocarcinoma, who was incorrectly classified as a non-responder by all the CNNs (false negative). Scale bar: 5 mm. B) Close-up view showing fragmented and stretched tumor glands. Scale bar: 250 μm. C) WSI of a non-responder patient affected by spindle cell carcinoma, who was incorrectly classified as a responder by all the CNNs (false positive). Scale bar: 2.5 mm. D) Close-up view showing tumor infiltrating the submucosal stroma. Scale bar: 250 μm.

Interestingly, all five tested CNNs correctly classified 10/23 (43.5%) patients, of whom 4 were responders and 6 were non-responders. On the other hand, all five the CNNs failed to correctly identify 2/23 patients; in both cases, the tissue samples were obtained from biopsies. We reviewed the original digital images for these patients, and found that one of these two cases exhibited few tumor cells and some tissue artifacts, suggesting that software analyses were biased by inadequate tissue samples (Fig 14).

### Comparison on models

It is important to note that the performance metrics may vary based on the dataset, training parameters, and hardware configurations employed. We summarize the performance and performance of the different models in our experiments.

A closer examination of Tables 3 and 4 reveals the architecture-specific accuracy metrics. Noteworthy is the prominently superior accuracy demonstrated by the GoogLeNet architecture. In contrast, the AlexNet and MobileNet configurations manifest the lowest levels of performance. While these initial scores might ostensibly suggest the limited efficacy of transfer learning for classification endeavors, an alternate interpretation is engendered by the subsequent section of Tables 3 and 4.

It can be seen that as for AlexNet, its exceptional accuracy upon its introduction thereby sets a foundational precedent for deep CNNs in the domain of image classification. Given its capacity to capture intricate features, AlexNet typically exhibits competitive F1 scores and precision, enabling reliable discrimination across classes. However, even though AlexNet introduced innovations in GPU parallelization, the model’s relatively larger parameter count could potentially impede efficient inference and training times.

Meanwhile, as for VGG, the uniform depth and consistent use of 3x3 convolutional filters in VGG contribute to elevated accuracy levels, attributed to the network’s heightened capability for intricate feature learning. The model’s capacity to develop comprehensive feature representations lends itself to commendable F1 scores and precision, indicating robust performance in classification tasks. VGG’s extensive depth, despite its accuracy benefits, often results in extended computation times during both training and inference stages.

MobileNet maintains moderate accuracy levels while emphasizing efficiency. However, it may exhibit a slight reduction in accuracy compared to more complex architectures due to its streamlined design. By Leveraging its lightweight architecture, MobileNet can achieve competitive F1 scores and precision, reflecting its ability to discern patterns across classes. What is noticeable, MobileNet is optimized for speed, rendering it suitable for real-time applications on resource-constrained platforms.

GoogLeNet’s distinctive inception modules, capturing features at varying scales, contribute to its high accuracy levels. The model’s multi-scale representation enhances its capacity for discerning intricate patterns. Given its diverse feature extraction capabilities, GoogLeNet is positioned to yield robust F1 scores and precision, signifying its proficiency in classification tasks. Despite its architectural complexity, GoogLeNet manages to maintain reasonable processing speed owing to parallelization strategies and efficient convolutions.

ResNet’s deep architecture and skip connections attribute to its high accuracy, as the model is adept at capturing complex patterns across a range of classes. Benefitting from its comprehensive feature learning capabilities, ResNet is well-equipped to yield strong F1 scores and precision, indicative of its efficacy in classification scenarios. ResNet strikes a balance between accuracy and speed; its deep architecture is counterbalanced by the presence of skip connections, which expedite training and convergence, enhancing overall efficiency.

**Fig 15:**
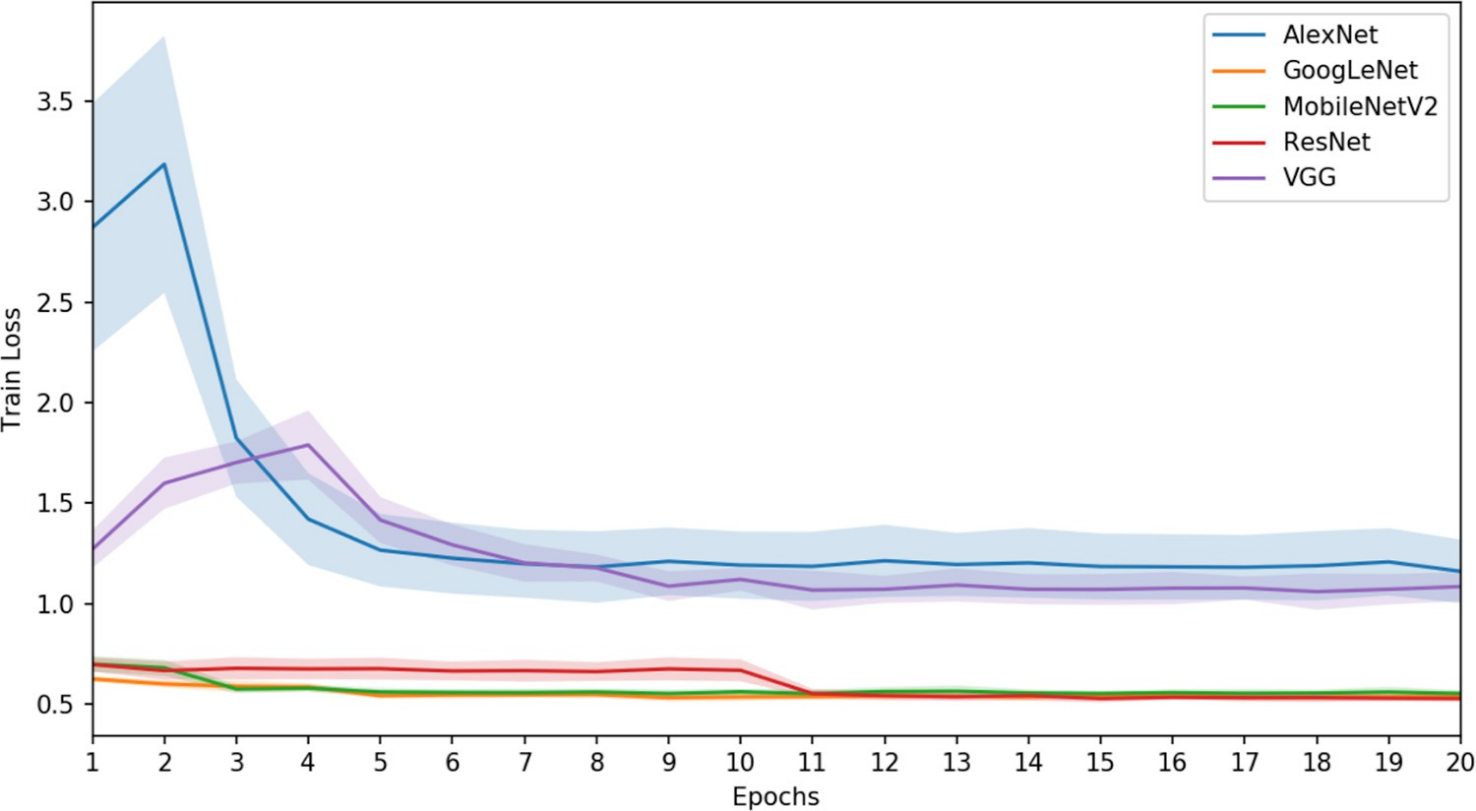
The evolution of training loss across epochs is delineated for the various CNN architectures. The solid line denotes the mean value encompassing the range of experiments, while the shaded colored region signifies the span of standard deviation.

Fig 15 depicts the training loss mean and standard deviation over the epochs for the diverse CNN architecture variations under scrutiny. A discernible trend across all architectures emerges, where the learning curve stabilizes after a modest number of training iterations. Notably, an exceptionality surfaces, wherein both the AlexNet and VGG architectures exhibit comparatively suboptimal training quality.

The GoogLeNet architecture continues to exhibit the most comprehensive performance metrics, attaining a commendable level of competence that underscores the potential viability of this approach. In stark contrast, echoing the patterns observed at the crop-level performances, the MobileNet configuration reflects the least favorable comprehensive scores at the patient level. Notably, the discernable performance advantage of GoogLeNet can be primarily ascribed to its adeptness at dimensionality reduction. This propensity enables the model to effectively capture and assimilate features even under conditions of limited available data. This characteristic, coupled with its judicious avoidance of overfitting, is instrumental in securing a high level of computational efficiency.

In synthesis, the manifested architectural variations in performance underscore the nuanced interplay between architecture design, transfer learning efficacy, and patient-level aggregation. This dynamic is notably captured by the GoogLeNet model, which exploits dimensionality reduction to effectively learn intricate patterns and achieve heightened performance without compromising computational efficiency.

### Pathomics analysis and tissue samples

#### Sample type

Considering the sample type, GoogLeNet seemed to be the best CNN for small tissue sample classification, correctly detecting 16/20 biopsies. With regards to surgical specimens, its success was lower, with 2/3 cases correctly classified. On the other hand, ResNet and MobileNet appeared to be more suitable CNNs for surgical specimens, each correctly classifying 3/3 samples. Moreover, regarding biopsies, 14/20 samples were correctly detected by ResNet, and 11/20 by MobileNet. AlexNet achieved successful classification of 15/20 biopsies, and 2/3 surgical specimens. VGG correctly classified 14/20 biopsies, and showed the worst results for surgical specimens, correctly classifying 0/3 samples.

#### Histological diagnosis

Considering the histological diagnosis, ResNet seemed to be most suitable for correctly predicting the response of patients affected by adenocarcinoma (12/14; 85.7%), compared to squamous cell carcinoma (5/9; 55.5%). With regards to squamous cell carcinoma, GoogLeNet yielded the best results, correctly identifying 8/9 (88.8%) patients. Although we observed some differences among CNNs in terms of predictive classification in adenocarcinomas vs squamous cell carcinomas, we did not find any statistically significant difference (AlexNet: p = 0.749, r = 0.71; GoogLeNet: p = 0.344, r = 0.207; MobileNet: p = 0.213, r = 0.270; ResNet: p = 0.118, r = 0.335; VGG: p = 0.458, r = 0.163).

## Discussion

Several studies have already explored ways to apply machine learning classifiers to pathology, with the aim of developing a CAD system. Notably, CNN-based approaches have been proposed for the classification of H&E-stained histological breast and gastric cancers [13, 15]. Some authors have also proposed CAD systems for detecting lymph node metastasis, or to automate the mitotic count [14, 16, 34]. Moreover, an AI system has been proposed for the automated Tumor Proportional Score (TPS) assessment of PD-L1 expression [35].

A few studies have tried to apply AI to histological images, with the goal of finding new image-based prognostic and predictive markers. In 2016, Kun-Hsing Yu et al. described a machine learning classifier designed to distinguish longer-term survivors from shorter-term survivors among patients with stage I NSCLC [17]. Xiangxue Wang et al. developed a system that could identify a high risk of recurrence among patients with early-stage NSCLC, with accuracy of between 75–82% [18]. In another study, Shidan Wang et al. described a high-risk group of patients with NSCLC, and found that image-based tumor shape features were an independent prognostic factor [19]. Rakaee Mehrdad et al. developed a machine-learning-based tumor-infiltrating lymphocyte (TIL) score that predicted the response of NSCLC to immune checkpoint inhibitor treatment [36]. Moreover, Nicolas Coudray et al. applied AI to digitalized tissue slides to predict the presence of mutations in lung adenocarcinoma [37]. Together, these findings suggest that H&E-stained slides potentially contain information that can be used to predict prognosis and/or treatment outcomes. The application of deep-learning techniques to histopathological images, to explore the prognostic and predictive potential of AI, has been defined as deep pathomics.

Among the five presently tested pre-trained CNNs (AlexNet, VGG, MobileNet, GoogLeNet, and ResNet), the best results in terms of specificity were obtained using GoogLeNet (TNr: 0.91), which correctly classified 10/11 non-responder patients. On the other hand, ResNet achieved the best results in terms of sensitivity (TPr: 0.75), correctly detecting 9/12 responder patients. Overall, we demonstrated that our deep pathomics approach was more suitable for identifying non-responder than responder patients.

All of the tested CNNs correctly classified 10/23 patients (4 responders and 6 non-responders), suggesting that some tumors exhibit specific morphological features that were easily detectable as responder or non-responder patients by all CNNs. On the other hand, 2/23 samples were incorrectly classified by all the CNNs. In future research, it will be interesting to define these specific features as “inclusion image-based criteria” for eligibility for deep pathomics analysis. Similarly, there are morphological inclusion criteria for eligibility for *transcriptomics* analysis (e.g., gene expression profiling), where the predictive value is meaningful only in cases with specific breast cancer features (e.g., ER+, HER2−, etc.) [38].

With regards to sample type (biopsies vs resected specimens), ResNet and MobileNet seemed to be more suitable for correctly classifying surgical specimens (3/3, 100%), while GoogLeNet achieved the best results among biopsies samples (16/20, 80%). Interestingly, since the best results were obtained from resected samples, we speculate that calculation of the predictive power of AI approaches should account for the tissue sample size. Moreover, we noted that some CNNs performed better in small samples rather than in surgical specimens. For example, VGG correctly classified 14/20 biopsies samples (70%), but failed to correctly identify all surgical resected specimens (0/3, 0%). These data support the hypothesis that the choice of specific CNN for deep pathomics analysis should be made based on the sample tissue type. Intriguingly, this concept resembles the situation in genomics analysis, where the analysis strategy should be guided by sample tissue type to achieve results of the highest accuracy [39].

Currently, predictive methods in NSCLC focus on target therapy or immunotherapy, rather than chemoradiation response. Molecular techniques such as NGS, PCR, and FISH, as well as immunohistochemical methods, are adopted to predict the response to target therapy and immunotherapy. However, these approaches have some limitations. Firstly, these methods require testing on FFPE tumor tissue samples. In advanced stages, NSCLC diagnoses are often based on small biopsies. Consequently, after diagnostic procedures, there might not be enough residual tissue for additional analyses. Secondly, it is important to note that not all patients with targetable drug mutations will respond to treatment. For instance, patients affected by lung adenocarcinoma are commonly screened for ALK rearrangements to identify potential candidates for ALK inhibitors (approximately 4% of NSCLC patients). Nevertheless, only around 60% of these patients will exhibit a positive response to the therapy [40].

**Fig 16:**
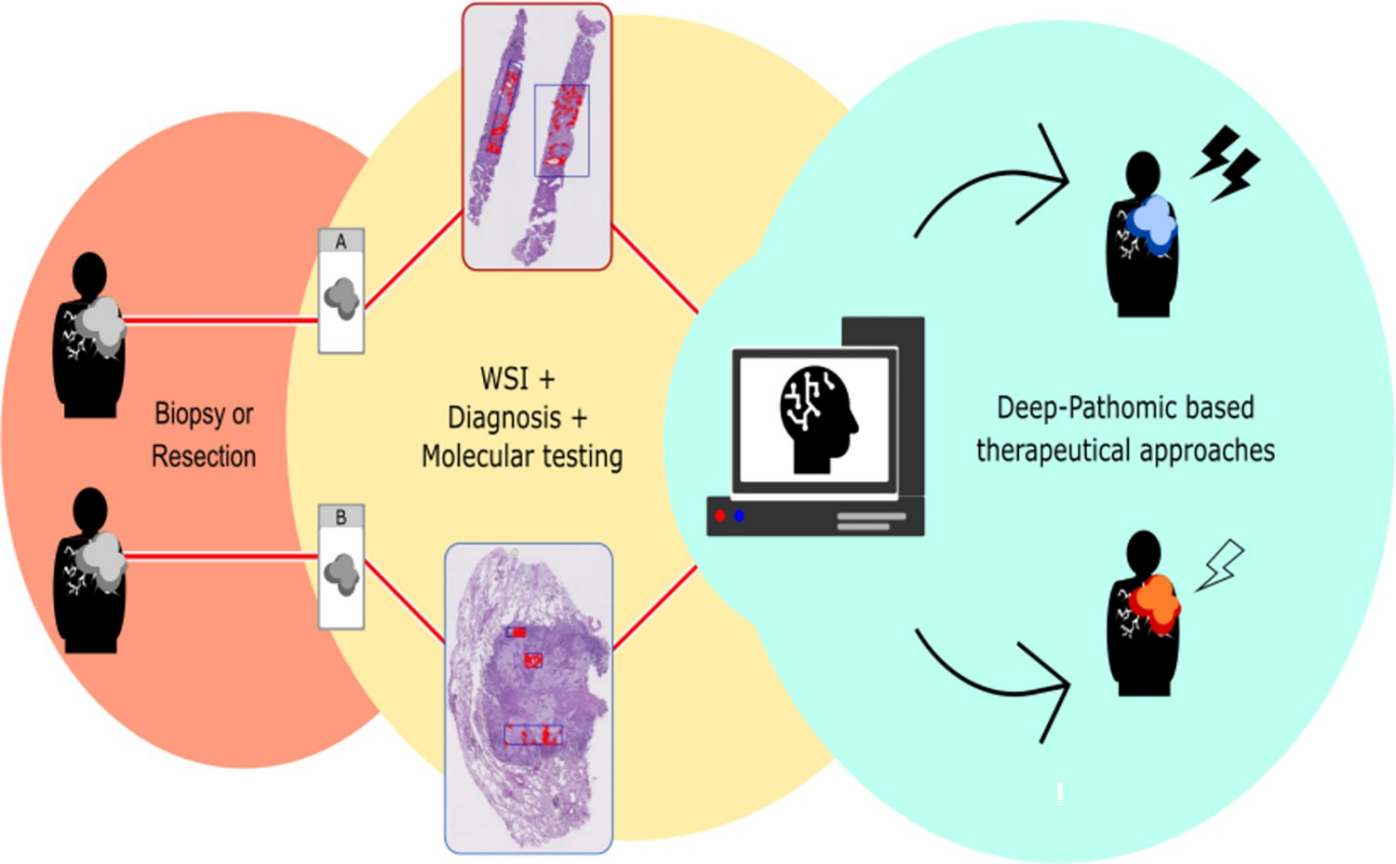
The flowchart outlines the potential practical implementation of deep-pathomics. Following tissue collection, either through biopsy or surgery, and subsequent histological diagnosis, the tumor tissue slides undergo digitalization via Whole Slide Imaging, without further additional tissue consuming; therefore, the residual tumor tissue will be available for molecular testing and immunohistochemistry. Tumor tissue slides from eligible patients for chemoradiation will undergo assessment using Deep-Pathomics. Based on this analysis, different personalized therapeutic strategies can be employed accordingly. The illustration depicts two distinct patients affected by Stage IIIB adenocarcinoma of the lung. Despite the same diagnosis, these patients underwent different treatment approaches as determined by Deep-Pathomics analysis.

In this context, Deep-Pathomics has emerged as a valuable resource. At first, predicting the response to chemoradiation therapy, it provided a missing data in NSCLC assessment; then, it revealed to be a highly sensitive analysis, if compared to the existing methods adopted for predicting responses to molecular target therapies. Importantly, Deep-Pathomics can be conducted alongside molecular testing without requiring additional tissue consumption and remains effective even when applied to small tissue biopsies. In Fig 16 we conjectured a hypothetical flow-chart to Deep-Pathomics applications.

In our previous experience, we identified that a specific radiomic signature extracted from CT images, mixing semantic and image-based features, was predictive of radiation response [41]. In the current study, we found that deep pathomics was a highly specific approach that was able to correctly detect almost all non-responder patients. The ability to predict response to treatment could guide the development of new and more effective therapeutic AI-based approaches. AI appears to exceed the capabilities of all presently available diagnostic systems, supplying additional information beyond the data currently provided in clinical practice.

## Conclusion

In current clinical practice, there is no way to predict response to chemoradiotherapy among patients with advanced-stage NSCLC. Therefore, in this study, we explored the capability of AI to predict oncological treatment response, through the analysis of digital images of H&E-stained tissue. We used five different CNNs to investigate patients with NSCLC treated with concurrent chemoradiation according to the current standard of care. Interestingly, we found that analysis precision could reach a score of 0.89.

The limitations of our present study were the small number of samples analyzed, the imbalance between sample types analyzed (31 biopsies and 4 resected specimens), and the retrospective cohort studied. Nevertheless, this is one of the first attempts to explore the potential use of a deep pathomics approach to predict chemoradiation treatment response. There remains a need for future data in a more extensive number of patients, and to validate the analysis in a prospective cohort.

As presented, even if applied on a still not exhaustive amount of subjects, Deep Learning Convolutive models have a true potential to be effectively applied in pathomics fields and therefore to improve the quality of medical outcome in terms of chemoradiotherapy response.

## Supporting information

S1 Table Classification category for each image by each network. Correct (TP and TN) and incorrect (FP and FN) calls of each convolutional neural network (CNN) for each patient from the test set (numbered from 1 to 23).
(DOCX)
