# Supplementary material for: Deep pathomics: A new image-based tool for predicting response to treatment in stage III non-small cell lung cancer
Source: PLoS One. 2023 Nov 28;18(11):e0294259. doi: 10.1371/journal.pone.0294259 (PMC10684067; doi:10.1371/journal.pone.0294259)
Supplement: S1 Table — Correct (TP and TN) and incorrect (FP and FN) calls of each convolutional neural network (CNN) for each patient from the test set (numbered from 1 to 23). (DOCX) [file pone.0294259.s001.docx]

**Table S1. Correct (TP and TN) and incorrect (FP and FN) calls of each convolutional neural network (CNN) for each patient from the test set (numbered from 1 to 23).**

| **Patient n°** | **AlexNet** | **GoogLeNet** | **MobileNet** | **ResNet** | **VGG** |
| --- | --- | --- | --- | --- | --- |
| 1 | TP | TP | TP | TP | TP |
| 2 | TN | TN | TN | TN | TN |
| 3 | TP | TP | TP | TP | TP |
| 4 | TN | TN | TN | TN | TN |
| 5 | FN | FN | TP | TP | FN |
| 6 | TN | TN | FP | TN | TN |
| 7 | TP | TP | TP | TP | FN |
| 8 | TN | TN | TN | TN | TN |
| 9 | TP | TP | FN | TP | TP |
| 10 | FN | TP | FN | FN | FN |
| 11 | FP | TN | FP | FP | TN |
| 12 | TP | TP | TP | TP | TP |
| 13 | TN | TN | TN | TN | TN |
| 14 | TP | TP | FN | FN | FN |
| 15 | TN | TN | FP | FP | TN |
| 16 | FN | FN | FN | TP | FN |
| 17 | TP | TP | TP | TP | TP |
| 18 | TN | TN | TN | TN | TN |
| 19 | FN | FN | FN | FN | FN |
| 20 | TN | TN | TN | TN | FP |
| 21 | TP | FN | TP | TP | TP |
| 22 | FP | FP | FP | FP | FP |
| 23 | TN | TN | TN | TN | TN |
| **Statistics** |  |  |  |  |  |
| **TP** | 8 | 8 | 7 | 9* | 6^¶^ |
| **FP** | 2 | 1* | 4^¶^ | 3 | 2 |
| **TN** | 9 | 10* | 7^¶^ | 8 | 9 |
| **FN** | 4 | 4 | 5 | 3* | 6^¶^ |
| **TPr** | 0.67 | 0.67 | 0.58 | 0.75* | 0.50^¶^ |
| **TNr** | 0.82 | 0.91* | 0.64^¶^ | 0.73 | 0.82 |

The lower section reports the true positive rate (TPr) and the true negative rate (TNr).

Green: true positive patients (TP); blue: true negative patients (TN); red: false positive patients (FP); yellow: false negative patients (FN).

* better results.

^¶^ worst results.
